# Code-Time Diversity for Direct Sequence Spread Spectrum Systems

**DOI:** 10.1155/2014/146186

**Published:** 2014-04-10

**Authors:** A. Y. Hassan

**Affiliations:** ^1^Benha Faculty of Engineering, Egypt; ^2^Faculty of Engineering, Northern Border University, Saudi Arabia

## Abstract

Time diversity is achieved in direct sequence spread spectrum by receiving different faded delayed copies of the transmitted symbols from different uncorrelated channel paths when the transmission signal bandwidth is greater than the coherence bandwidth of the channel. In this paper, a new time diversity scheme is proposed for spread spectrum systems. It is called code-time diversity. In this new scheme, *N* spreading codes are used to transmit one data symbol over *N* successive symbols interval. The diversity order in the proposed scheme equals to the number of the used spreading codes *N* multiplied by the number of the uncorrelated paths of the channel *L*. The paper represents the transmitted signal model. Two demodulators structures will be proposed based on the received signal models from Rayleigh flat and frequency selective fading channels. Probability of error in the proposed diversity scheme is also calculated for the same two fading channels. Finally, simulation results are represented and compared with that of maximal ration combiner (MRC) and multiple-input and multiple-output (MIMO) systems.

## 1. Introduction

Diversity techniques are used when the channel is in a deep fade. If several replicas of the same information signal are transmitted over independent fading channels, the probability that all signal components will fade simultaneously is reduced considerably. There are different ways in which we can provide the receiver with *L* independent fading replicas of the same information signal.

Frequency diversity is a diversity method where the information signal is transmitted on *L* carriers. The separation between the successive carriers equals to or exceeds the coherent bandwidth of the channel. Orthogonal frequency division multiplexing (OFDM) transmission is the famous technique that exploits frequency diversity to achieve high data rate and low bit error rate in frequency selective channels [1–3]. OFDM suffers from intercarrier interference (ICI) due to frequency offsets, symbol timing error, and channel estimation errors [4–6]. OFDM systems also suffer from high peak to average power ratio (PAPR) [7, 8].

Another commonly used method for achieving diversity employs multiple antennas. Multiple transmitting antennas are used to transmit the same information signal and multiple receiving antennas are used to receive the independently fading replicas of the transmitted signal through uncorrelated fading paths. A comparative study of space diversity techniques in mobile radio is shown in [9]. Multiple-input and multiple-output (MIMO) system is a well-known system that exploits the antenna diversity to enhance the bit error rate and the channel capacity in fading environments [10, 11].

Time diversity is another diversity method where *L* independent fading version of the same information signal is achieved by transmitting the signal in *L* different time slots. The separation between the successive time slots equals to or exceeds the coherence time of the channel. Time diversity is used in modern communication system through interleaving of the transmitted symbols and through the using of channel codes [12–14].

Some systems use a combination of more than one diversity technique to enhance their performance in fading channels such as space-time (ST) coding in MIMO systems [15, 16], which use the time and space diversities through encoding the transmitted symbols using space time codes and transmitting the encoded symbols by different antennas in the transmitter. This technique allows the data symbol to be transmitted more than one time through different symbol period and it arrives at the receiver's antennas through different spatial paths. MIMO-OFDM system is another example of multidiversity system where time, frequency, and space diversities are used to enhance the performance of the data transmission over wireless faded channels [17–20]. In MIMO-OFDM system, the symbols are encoded by using space-time-frequency (STF) block codes. The encoded symbol is transmitted more than once over different periods and carrier frequencies using different transmitting antennas. The uncorrelated fading gains that come from the uncorrelated spatial paths, the different transmitting time slots, and the different transmitting carrier achieve the diversity gain at the receiver. This system has large diversity gain and better performance than space-time coded MIMO systems.

The systems that use space diversity such as MIMO systems have the disadvantage of using more than one antenna at the transmitter and the receiver. Multiple antennas need multiple RF drivers (power amplifier at the transmitter and low noise amplifier at the receiver) and this complicates the transmitter and the receiver structure. The spacing between the antennas should be large enough to have uncorrelated fading paths and to reduce the cross correlation and interference between the antennas. The MIMO systems consume more power than single-input and single-output (SISO) systems and they have short lifetime batteries for mobile units. Powerful DSP unit is required for MIMO transceivers because ST and STF encoder and decoder need complex computations.

In this paper, we propose a new diversity technique for SISO spread spectrum systems that can achieve a diversity gain like that of the MIMO system but with a single transmitting antenna and a single receiving antenna. The MIMO system has diversity gain with two degrees of freedom (the number of the transmitting and receiving antennas). The proposed diversity scheme has a diversity gain with two degrees of freedom too. Although the proposed system has a single transmitting antenna and a single receiver antenna, the using of *N* spreading codes and *L* uncorrelated propagation paths achieves the diversity gain.  Section 2 discusses the idea of code-time diversity in detail. In Section 3 the signal model and the new transmit diversity scheme are represented. The receiver structure of the proposed system is shown in Section 4 with the calculations of the probability of error in the received data.  Section 5 shows the simulation results and some implementation issues.

## 2. Code-Time Diversity

In space-time MIMO system, the encoded data symbol is transmitted more than one time through different symbol periods using different transmitting antennas. The transmission of the symbols through independent time slots lets the received symbol to have independent fading gains. The probability of receiving the transmitted symbol with faded gains through the successive time slots is reduced significantly. Also the usage of different antennas allows the transmitted symbol to have independent propagation paths from the transmitter to the receiver and to have independent fading gain through each path. So they are the different time slots and propagation paths that play the main role in the diversity gain enhancement of the time-space MIMO system.

**Figure 1:**
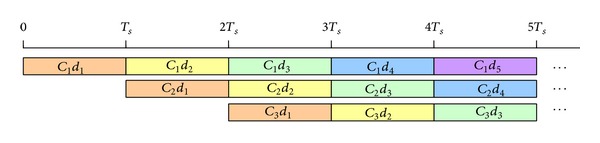
The code-time diversity system with 3 orthogonal spreading codes.

In the proposed code-time diversity technique, we use the same concept of time and space diversities but through another procedure. During each symbol period, the current data symbol and the previous (*N* − 1) ones are dispersed in frequency using *N* independent spreading codes sequences for each data symbol. The used spreading codes are taken from a set of *N* orthogonal codes. The dispersed symbols are added together and transmitted using a single antenna. The orthogonality between the used spreading sequences prevents the interference among the transmitted symbols. The same procedure is repeated for each symbol period so that each symbol can be transmitted *N* times through *N* successive symbol periods using, each time, a different spreading code from the set of *N* orthogonal codes.  Figure 1 shows an example of how the modulated data symbols are transmitted three times at three successive symbol periods using three different orthogonal spreading codes.

By this way, time diversity is achieved and the probability of having *N* faded gains during *N* successive symbols periods is reduced considerably.

The space diversity is achieved by controlling the bandwidth of the dispersed symbols to be greater than the coherent bandwidth of the used wireless channel. This allows uncorrelated multipath propagation from the transmitter to the receiver. The using of direct sequence spread spectrum (DSSS) increases the information message bandwidth by a factor equal to the process gain of the spreading process, which is equivalent to the ratio between the data symbol period and the spreading code chip period. By controlling the process gain, the bandwidth of the spread signal can be equal to multiples of the coherent bandwidth of the channel. The number of the uncorrelated paths that can be appeared from the transmitter to the receiver is given by

(1) $$\begin{array}{c} L=\left\lfloor \frac{\text{Bandwidth of DSSS signal}}{\text{Channel coherent bandwidth}}+0.5\right\rfloor . \end{array}$$

By the same way, the probability of having spatial faded gains through the uncorrelated propagation paths *L* is reduced significantly.

The proposed code-time diversity system has a diversity gain with two degrees of freedoms as the space-time MIMO system. Although the diversity gain in the time-space MIMO system is depending on the number of the antennas in the transmitter *N*_*t*_ and the receiver *N*_*r*_, the diversity gain in the code-time diversity system is depending on the number of the used spreading codes *N* (which equals to the number of time slots through which each symbol will be repeated) and the number of the uncorrelated propagation paths *L*. The proposed diversity scheme uses one antenna and one RF interface unit in the transmitter and in the receiver. However, the code-time diversity seems to have diversity gain similar to that of the time-space MIMO system, and the code-time diversity uses a spread signal with higher bandwidth than the bandwidth of the transmitted signal in the time-space MIMO system. In other words, the increase in the signal bandwidth of the proposed code-time diversity system is the cost that should be paid to improve the diversity gain using a simplified hardware of single antenna and single RF interface in the transmitter and in the receiver. The code-time diversity system needs no space-time codes, and it depends only on the orthogonality between the spreading codes.

## 3. Transmitted Signal Model of Code-Time Diversity DSSS 

The code-time diversity system is based on the transmission of the data by using different orthogonal spreading codes at different transmission periods. At each transmission period, the transmitted signal is the summation between the current spread symbol and the previous (*N* − 1) spread symbols where *N* is the number of the used orthogonal spreading codes. Equation (2) shows the *k*th symbol transmission:

(2) $$\begin{array}{c} s_{k}\left(t\right)=\overset{N-1}{\underset{n=0}{\sum }}d_{k-n}c_{n}\left(t-kTs\right), \end{array}$$

where *d*_*k*−*n*_ is the modulated data symbol and *c*_*n*_(*t*) is the spreading code sequence. The used modulation method may be BPSK or *M*-QAM. The spreading codes are assumed to be orthonormal through the symbol period *T*_*s*_:

(3) $$\begin{array}{c} \overset{T_{s}}{\underset{0}{\int }}c_{i}\left(t\right)\cdot c_{j}\left(t\right)\cdot dt=\{\begin{array}{cc} 1 & \text{if }i=j\\ 0 & \text{if }i\neq j. \end{array} \end{array}$$

Without any loss of generality, the code period is assumed to be equal to the symbol period:

(4) $$\begin{array}{c} T_{s}=N_{c}*T_{c}. \end{array}$$

*N*_*c*_ is the number of chips on one code period and *T*_*c*_ is the chip period. So,

(5) $$\begin{array}{c} c_{i}\left(t-kT_{s}\right)=c_{i}\left(t\right). \end{array}$$

**Figure 2:**
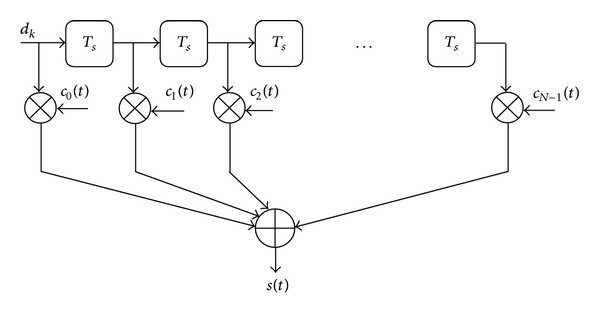
The code-time diversity DSSS modulator.

The transmitted signal of a packet of *K* symbols is illustrated in (6) and Figure 2 shows the modulator structure of code-time diversity system:

(6) $$\begin{array}{c} s\left(t\right)=\overset{K-1}{\underset{k=0}{\sum }}s_{k}\left(t-kTs\right)=\overset{K-1}{\underset{k=0}{\sum }}\;\overset{N-1}{\underset{n=0}{\sum }}d_{k-n}c_{n}\left(t-kTs\right). \end{array}$$

The modulated signal in (6) is transmitted to the channel through single RF interface module and single transmitting antenna.

## 4. Received Signal Model of Code-Time Diversity DSSS Signal in Rayleigh Fading Channel and the Proposed Demodulator Building

### 4.1. Flat Fading Rayleigh Channel

The impulse response of the flat fading channel is shown in (7). Quasistatic channel is assumed where the fading gain is fixed during one symbol period and it is changed randomly from one symbol to another:

(7) $$\begin{array}{c} h\left(t\right)=\alpha \delta \left(t\right), \end{array}$$

where *α* is a complex Gaussian random variable with zero mean and *σ*_*α*_^2^ variance. In flat fading channel, the transmitted signal travels from the transmitter to the received through unresolvable propagation paths. Therefore, all frequency components of the signal will experience the same magnitude of fading. In our proposed diversity scheme, the flat fading case looks like the MISO system where multiple antennas transmit the modulated symbols and a single antenna at the receiver picks them up. According to our signal model, the received signal at the demodulator input is

(8) $$\begin{array}{c} r\left(t\right)=\overset{K-1}{\underset{k=0}{\sum }}\;\overset{N-1}{\underset{n=0}{\sum }}{\alpha_{k}d}_{k-n}c_{n}\left(t-kTs\right)+w\left(t\right), \end{array}$$

where *w*(*t*) is a sample function of white Gaussian noise process with zero mean and *σ*_*w*_^2^ variance. *α*_*k*_ is the channel random gain at the *k*th symbol period. The proposed demodulator for the code-time diversity system consists of three parts. The first part is a bank of correlators that correlate the received signal with the *N* spreading codes. The *n*th correlator correlates the received signal with the *n*th spreading code *c*_*n*_(*t*) through one symbol period. The *n*th correlator output at the *k*th symbol period is shown in (9a) and (9b):

(9a) $$\begin{array}{c} x_{n}\left(kT_{s}\right)=\overset{T_{s}}{\underset{0}{\int }}r\left(t\right)\cdot c_{n}\left(t-kT_{s}\right)\cdot dt={\alpha_{k}d}_{k-n}+v_{kn}, \end{array}$$

(9b) $$\begin{array}{c} v_{kn}=\overset{T_{s}}{\underset{0}{\int }}w\left(t\right)\cdot c_{n}\left(t-kT_{s}\right)\cdot dt, \end{array}$$

where *v*_*kn*_ is a Gaussian random variable with zero mean and *σ*_*w*_^2^ variance. The second part of the proposed demodulator is the combiner. Maximal ration combiner (MRC) is used but with some modifications. In the proposed MRC, the outputs of the *N* correlators are multiplied by the conjugate of the channel gain *α*_*k*_, which is estimated in the receiver. The multiplications results will independently be delayed according to the spreading code index. The output of the correlation with the code sequence *c*_*n*_(*t*) is delayed ((*N* − 1) − *n*) symbol periods. The delayed samples are finally added to form a single input to the detector. The output of the proposed MRC in the *z*-domain can be represented by

(10) Y(z)=β0X0(z)z−(N−1)+β1X1(z)z−(N−1)+1 +β2X2(z)z−(N−1)+2+⋯+βN−1XN−1(z).

The *β*_*n*_ coefficients represent the conjugate of the estimated channel gains according to the following relation:

(11) $$\begin{array}{c} \beta_{n}\left(kT_{s}\right)=\alpha_{k-\left(N-1\right)+n}^{*}. \end{array}$$

The output of the combiner at the *k*th symbol period is represented by

**Figure 3:**
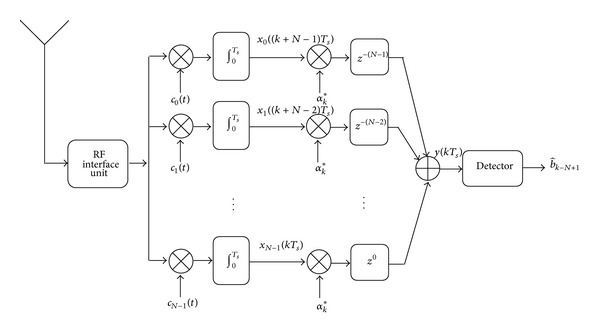
The code-time diversity DSSS receiver in Rayleigh flat fading channel.

(12) y(kTs)=∑n=0N−1αk−(N−1)+n∗xn((k−(N−1)+n)Ts)=∑n=0N−1αk−(N−1)+n∗αk−(N−1)+ndk−(N−1)+vkn′,

(13a) $$\begin{array}{c} y\left(kT_{s}\right)=\overset{N-1}{\underset{n=0}{\sum }}{\left|\beta_{n}\left(kT_{s}\right)\right|}^{2}d_{k-(N-1)}+v_{kn}^{\prime },\;\;\;\;\;\;\; \end{array}$$

(13b) $$\begin{array}{c} v_{kn}^{\prime }=\overset{N-1}{\underset{n=0}{\sum }}\beta_{n}\left(kT_{s}\right)v_{k-(N-1)+n},\;\;\;\;\;\;\; \end{array}$$

where *v*_*kn*_′ is a Gaussian random variable with zero mean and ∑_*n*=0_^*N*−1^|*β*_*n*_(*kT*_*s*_)|^2^ *σ*_*w*_^2^ variance.  Figure 3 shows the receiver block diagram for the code-time diversity scheme. The estimated data at the output of the detector is late (*N* − 1) symbol periods. This delay represents the time spread at which the transmitted symbol is repeated.

The last part of the demodulator is the detector. The optimum detector computes the Euclidian distance between the received symbol and all the symbols in the symbols constellation diagram. The detector decides that *d*_*i*_ is transmitted if and only if the distance between *y*(*kT*_*s*_) and *d*_*i*_ is smaller than the distance between *y*(*kT*_*s*_) and *d*_*m*_ for all *m*:

(14) choose  di⟺d2〈y(kTs),di〉<d2〈y(kTs),dm〉∀i≠m.

The combined signals in (12) are equivalent to that of *N*-branch MRC receiver. Therefore, the resulting diversity order from the new code-time diversity scheme with *N* spreading orthogonal codes and one transmission antenna is equal to that of the *N*-branch MRC receiver scheme.

It is important to emphasize on that the combined signals in (12) are similar to that of space-time MIMO system with *N* antennas at the transmitter and one antenna at the receiver or a space-time MIMO system with one antenna at the transmitter and *N* antennas at the receiver. The proposed code-time diversity system does not use additional encoders or decoders at the transmitter or the receiver such as the space-time encoder and decoder in the space-time MIMO system. No additional RF interface circuits or antennas are used in the code-time diversity. Spreading and dispreading circuits are the only used additional hardware. The disadvantage of the proposed code-time diversity system is the extended bandwidth used and the *N* symbol period delays that precede the detection of the first transmitted symbol.

*Probability of Symbol Error.* To determine the probability of symbol error in the proposed code-time diversity system, the decision variable is calculated first. The optimum detector calculates the decision variable by multiplying the signal in (12) with the conjugate of all the complex symbols on the constellation diagram. The estimated symbol is the symbol with the largest decision variable. If symbol *i* is the symbol transmitted at the *k*th symbol period, the largest decision variable will be

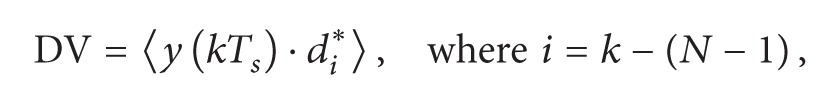
(15)

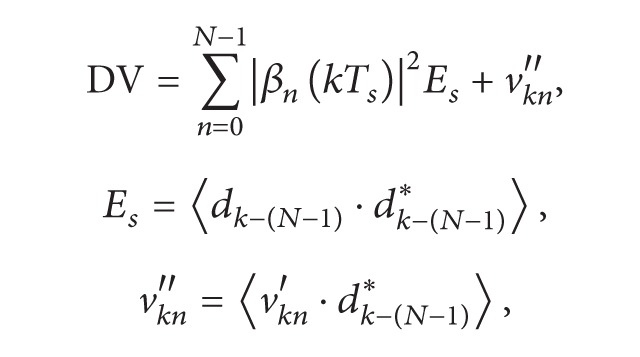
(16)
where *v*_*kn*_′′ is a Gaussian random variable with zero mean and ∑_*n*=0_^*N*−1^|*β*_*n*_(*kT*_*s*_)|^2^ *σ*_*w*_^2^ *E*_*s*_ variance. Therefore, the decision variable (DV) in (16) is also a Gaussian random variable with the following mean and variance:

(17a) $$\begin{array}{c} E\left[\text{DV}\right]=\overset{N-1}{\underset{n=0}{\sum }}{\left|\beta_{n}\left(kT_{s}\right)\right|}^{2}E_{s}, \end{array}$$

(17b) $$\begin{array}{c} \mathrm{Var}\left[\text{DV}\right]=\overset{N-1}{\underset{n=0}{\sum }}{\left|\beta_{n}\left(kT_{s}\right)\right|}^{2}\sigma_{w}^{2}E_{s}. \end{array}$$

According to the used modulation method, the probability of symbol error is always a function of the signal to noise ratio. This probability of error will be a random variable because the signal to noise ratio is a random variable. The instantaneous SNR is represented as shown in

(18) $$\begin{array}{c} \text{SNR}\left(kT_{s}\right)=\frac{\sum_{n=0}^{N-1}{\left|\beta_{n}\left(kT_{s}\right)\right|}^{2}E_{s}}{{2\sigma }_{w}^{2}}, \end{array}$$

where |*β*_*n*_(*kT*_*s*_)|^2^ is a chi-square random variable. Thus, the conditional probability of symbol error is calculated first for a certain value of the SNR, and then the average probability of error is calculated by averaging the conditional probability of symbol error over the probability density function of the SNR. For *M*-QAM, the probability of symbol error is given by

(19) PQAM=4(1−1M)Q() −4(1−1M)2Q2(3M−1∑n=0N−1|βn(kTs)|2Es2σw2).

For simplicity (19) can be approximated by the first term only so that the *Q* ^2^(*x*) is ignored since *Q* ^2^(*x*)≪*Q*(*x*) [21]. In the case of nonfading channel, the probability of symbol error in code-time diversity system is

(20) $$\begin{array}{c} P_{\text{QAM}}=4\left(1-\frac{1}{\sqrt{M}}\right)Q\left(\sqrt{\left(\frac{3}{M-1}\right)\frac{{NE}_{s}}{{2\sigma }_{w}^{2}}}\right). \end{array}$$

For fading channel, the probability density function of the signal to noise ratio is equal to the probability density function (pdf) of a chi-square random variable with 2*N* degrees of freedom as shown in

(21) p(SNR(kTs))  =1(N−1)!SNR−N(SNR(kTs))N−1e−(SNR(kTs))/SNR−.

$\overset{-}{\text{SNR}}$ is the average signal to noise ratio per time slot or by diversity channel and it is given by

(22) $$\begin{array}{c} \overset{-}{\text{SNR}}=\frac{E_{s}}{{2\sigma }_{w}^{2}}E\left[{\left|\beta_{n}\left(kT_{s}\right)\right|}^{2}\right]=\frac{\sigma_{\alpha }^{2}E_{s}}{2\sigma_{w}^{2}}. \end{array}$$

The average probability of symbol error is

(23) P−QAM=∫0∞PQAM·p(SNR(kTs))·dSNR=4(1−1M)(1(N−1)!SNR−N) ×∫0∞Q(3M−1SNR)·SNRN−1e−SNR/SNR−·dSNR.

After some mathematical manipulations, the exact value of the average probability of symbol error ${\overset{-}{P}}_{\text{QAM}}$ in code-time diversity is given by

(24) P−QAM=  4(1−1M)(1(N−1)!SNR−N) ×(2(M−1)3)NΓ(N+1/2)π(2N) ×2F1(N,2N+12;N+1;−2(M−1)3SNR−).

_*p*_ *F*_*q*_ is the generalized hyper-geometric function. It is defined in Appendix A.

### 4.2. Limitations of the Used Spreading Codes

The use of DSSS in the proposed code-time diversity system expands the transmitted signal bandwidth more than the bandwidth of the nonspread modulated symbols and also more than the bandwidth of the transmitted signal if a space-time coding MIMO system is used. Although the enlarged bandwidth in the code-time diversity system increases the channel capacity and increases the system resistance to jamming and interference signals, bandwidth efficiency of code-time diversity system is poor.

In order to increase the bandwidth efficiency in code-time diversity system, more than one user are allowed to share the same channel bandwidth but with a different set of orthogonal spreading codes. If *M* users share the same channel using the proposed diversity system, *M* × *N* orthogonal spreading codes are required. This increases the demands on the orthogonal spreading codes.

On the other hand, we can merely assign one PN sequence for each user in the multiuser code-time diversity system and by exploiting the autocorrelation property of the PN sequence, and the rest (*N* − 1) spreading codes required for the proposed diversity scheme can be generated from the same generator polynomial by cyclically shifting the generated sequence different (*N* − 1) times. For PN sequence with significant long period, the correlation between the generated PN sequence and its cyclically shifted sequences is very small but not zero. From [21], if *c*(*n*) is a PN sequence of period *N*_*c*_, the autocorrelation between this sequence and its cyclic shift with *n*_*i*_ chips is equals to

(25) 1Nc∑m=0Nc−1c(m)·c(m−ni)  ={1∀ni=0,Nc,2Nc,3Nc,…−1Ncelse where.

The use of multiuser DSSS system with code-time diversity enhances the bandwidth efficiency of the system, but in this case a multiuser detector should be used in the receiver shown in Figure 3 instead of a single user detector. This point will be discussed in detail in a separate research, but now we continue with a single user detector case.

Equations (20) and (24) show the probability of error and the average probability of error in the received data for the case of nonfaded and faded channels, respectively, assuming that the used *N* spreading codes are mutually orthogonal. On the other hand if nonorthogonal codes are used such as a PN sequence and its cyclic shifted sequences, the correlation between the codes pairs affects the probability of error. This correlation gives rise to the intersymbol interference (ISI) between the transmitted symbols.

In nonorthogonal spreading code case, the output of each correlator with each spreading code consists of the desired signal and (*N* − 1) interference signals from the previous and proceeding transmitted symbols. The output of the proposed MRC (*y*(*kT*_*s*_)) will have interference signals from the previous (*N* − 1) transmitted symbols and interference signals from the proceeding (*N* − 1) transmitted symbols. The *n*th correlator output at the *k*th symbol period is illustrated in

(26) xn(kTs)=∫0Tsr(t)·cn(t−kTs)·dt=αkdk−n+∑m=0m≠nN−1αkρnmdk−m+vkn.

The first term in the right hand side is the desired signal, the middle term is the ISI from the previous and proceeding symbols according to the value of *n*, and the last term is the Gaussian noise component. *ρ*_*nm*_ is the correlation between the spreading codes of index *n* and *m*:

(27) $$\begin{array}{c} \rho_{nm}=\overset{T_{s}}{\underset{0}{\int }}c_{n}\left(t-kT_{s}\right)\cdot c_{m}\left(t-kT_{s}\right)\cdot dt. \end{array}$$

According to (26), the output of the proposed MRC will be

(28) y(kTs)=∑n=0N−1|βn(kTs)|2dk−(N−1) +∑n=0N−1∑m=0m≠nN−1|βn(kTs)|2ρnmdk−(N−1)+n−m+vkn′.  

The decision variable of the optimum detector in Figure 3 will be

(29) DV=y(kTs)·dk−(N−1)∗=∑n=0N−1|βn(kTs)|2Es +∑n=0N−1 ∑m=0m≠nN−1|βn(kTs)|2ρnmdk−(N−1)+n−mdk−(N−1)∗+vkn′′.

The decision variable in (29) is a complex random variable. The detector will make its decision according to the real part of this random variable. The transmitted symbols are almost uncorrelated, so that the mean value of the decision variable can be represented by

(30) $$\begin{array}{c} E\left[\text{DV}\right]=\overset{N-1}{\underset{n=0}{\sum }}{\left|\beta_{n}\left(kT_{s}\right)\right|}^{2}E_{s}.\;\;\;\;\; \end{array}$$

The interference component in (29) can be treated as a noise signal added to the Gaussian noise component *v*_*in*_′′. The noise signal and the interference signals have zero means and they are uncorrelated; thus the variance of their summation is equal to the summation of their individual variances. The variance of the noise is represented in (17b). The variance of interference signal is

(31) Var⁡[∑n=0N−1∑m=0m≠nN−1|βn(kTs)|2ρnmdk−(N−1)+n−mdk−(N−1)∗]  =Es2∑n=0N−1∑m=0m≠nN−1|βn(kTs)|4ρnm2.

The noise and interference variance will be

(32) Var⁡[∑n=0N−1∑m=0m≠nN−1|βn(kTs)|2ρnmdk−(N−1)+n−mdk−(N−1)∗+vkn′′  ]  =Es2∑n=0N−1∑m=0m≠nN−1|βn(kTs)|4ρnm2+Esσw2∑n=0N−1|βn(kTs)|2.

The cross-correlation between a PN sequence and any of its shifted versions is constant and independent on the shift value as long as the shift is not zero or integer multiples of the code period. So, the noise and interference variance in (32) can be simplified to

(33) Var⁡[∑n=0N−1∑m=0m≠nN−1|βn(kTs)|2ρnmdk−(N−1)+n−mdk−(N−1)∗+vkn′′  ]  =(N−1)ρ2Es2∑n=0N−1|βn(kTs)|4+Esσw2∑n=0N−1|βn(kTs)|2.

From (30) and (33), the signal to interference and noise ratio SINR(*kT*_*s*_) is a random variable and its instantaneous value is defined by

(34) SINR(kTs) =(E[DV])22Var⁡[DV] =Es(∑n=0N−1|βn(kTs)|2)22(N−1)ρ2Es∑n=0N−1|βn(kTs)|4+2σw2∑n=0N−1|βn(kTs)|2.

The average probability of error will be

(35) PQAM=E[4(1−1M)Q×((3×(M−1)−1)×(Es(∑n=0N−1|βn(iTs)|2)2×(2(N−1)ρ2Es∑n=0N−1|βn(iTs)|4+2σw2∑n=0N−1|βn(iTs)|2)−1)1/2)].

**Figure 4:**
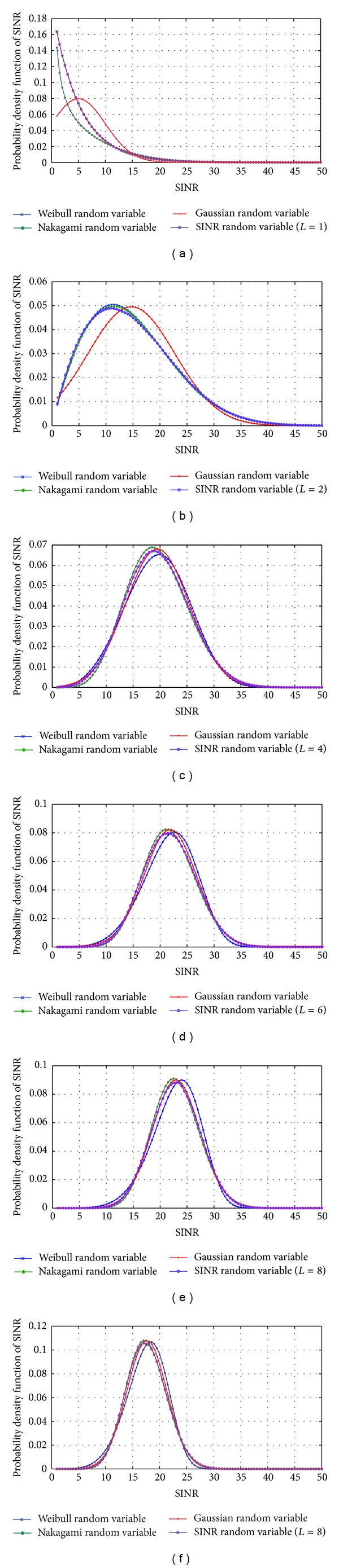
Comparison between the pdf of SINR random variable and the pdf of Weibull, Nakagami, and Gaussian random variables at different diversity orders (*L* = *N*).

The calculation of the exact value of the pdf of the SINR random variable in (35) is so difficult. So, another procedure is followed. The unknown pdf of SINR is calculated and plotted numerically using a lot of random samples of the SINR random variable (more than 10^6^ random samples). The plotted pdf is compared with the other pdf functions of some well-known random variables such as Weibull, Nakagami, and Gaussian random variables. After a lot of trials, it is found that the unknown pdf of the SINR random variable in (34) is very close to the pdf of Nakagami random variables as illustrated in Figure 4.

The figure contains the pdf of Weibull, Nakagami, and Gaussian random variables that are calculated according to the statistical averages of the samples of the SINR. At low average SINR, the unknown pdf is very close to the Nakagami pdf, but at high average SINR it will be closer to the Gaussian pdf since the Nakagami pdf comes closer to the pdf of the Gaussian random variable at high average SINR too. Increasing the diversity order increases the average SINR; thus at high diversity order, the unknown pdf of SINR can be approximated by the pdf of the Gaussian random variable. This result matches the center limit theory of random variables. Although the unknown pdf of SINR is close to Gaussian pdf at high average SINR, Nakagami pdf will be used to approximate this unknown pdf since Nakagami pdf gives a good approximation of the unknown pdf of SINR at low and high average values of SINR. Equation (36) is the probability density function of a Nakagami random variable:

(36) $$\begin{array}{c} p_{\text{SINR}}\left(\text{SINR}\right)=\frac{2}{\Gamma \left(m\right)}\cdot {\left(\frac{m}{\Omega }\right)}^{m}{\text{SINR}}^{2m-1}e^{-(m/\Omega ){\text{SINR}}^{2}},\;\;\; \end{array}$$

where *m* is the shape parameter and *Ω* is the scale parameter. The shape and scale parameters are related to the mean and the variance of the SINR random variable [22]:

(37) $$\begin{array}{c} m=\left(\frac{{\left(E\left[{\text{SINR}}^{2}\right]\right)}^{2}}{\text{var}\left[{\text{SINR}}^{2}\right]}\right),\;\;\Omega =E\left[{\text{SINR}}^{2}\right].\;\;\;\;\;\;\; \end{array}$$

Now, the average probability of error will be

(38) P−QAM  =∫0∞PQAM·p(SINR(kTs))·dSINR  =2(1−1M)2Γ(m)·(mΩ)m   ×∫0∞erfc⁡(32(M−1)SINR)     ×SINR2m−1e−(m/Ω)SINR2·dSINR,

(39) P−QAM =(1−1M)21−mΓ(2m)Γ(m)  ·(13·e((9/(32(M−1)2)·(Ω/m))·D−2m(98(M−1)2·Ωm)+e((1/(2(M−1)2)·(Ω/m))·D−2m(2(M−1)2·Ωm)).

*D*_*p*_(*z*) is the parabolic cylindrical function defined in [23]. The complete derivation of the average probability of symbol error in (39) is represented in Appendix B. If Gaussian pdf is used to model the pdf of SINR in (34), the average probability of symbol error will be

(40) P−QAM=2(1−1M) ·(16e(((9σ2)/(8(M−1)2))  −(3μ/2(M−1)))+12e(((2σ2)/(M−1)2)  −(2μ/(M−1)))),

(41) $$\begin{array}{c} \mu =E\left[\text{SINR}\right],\;\;\sigma^{2}=E\left[{\left(\text{SINR}-\mu \right)}^{2}\right]. \end{array}$$

### 4.3. Multipath Rayleigh Channel

The impulse response of the multipath fading channel is shown in (42). Quasistatic channel is also assumed where the fading gain *α*_*l*_ of the *l*th path is fixed during one symbol period and it is changed randomly from one symbol to another:

(42) $$\begin{array}{c} h\left(t\right)=\overset{L-1}{\underset{l=0}{\sum }}\alpha_{l}\delta \left(t-\tau_{l}\right), \end{array}$$

where *α*_*l*_ is a complex Gaussian random variable with zero mean and *σ*_*α*_*l*__^2^ variance. *τ*_*l*_ is the *l*th path delay. *L* is the number of the uncorrelated fading paths from the transmitter to the receiver. The frequency components of the signal will experience different magnitudes of fading. In our proposed diversity scheme, the multipath fading case looks like the MIMO system where multiple antennas transmit the modulated symbols and multiple antennas at the receiver picked them up. According to our signal model, the received signal at the demodulator input is

(43) $$\begin{array}{c} r\left(t\right)=\overset{K-1}{\underset{k=0}{\sum }}\;\overset{N-1}{\underset{n=0}{\sum }}\;\overset{L-1}{\underset{l=0}{\sum }}{\alpha_{kl}d}_{k-n}c_{n}\left(t-kTs-\tau_{l}\right)+w\left(t\right).\;\;\; \end{array}$$

As shown in (1), the number of the paths *L* depends on the bandwidth of the DSSS signal and the coherence bandwidth of the channel. By increasing the process gain of the DSSS system, the number of the uncorrelated propagation paths is increased.

Here a new important note should be mentioned. The idea of the proposed diversity scheme is based on transmitting each symbol through more than one symbol period using separate spreading codes. As shown in the previous section, a PN code with different cyclic shifts may be used to encode each data symbol. In multipath fading channel, improper choice of the different shift values increases the ISI because the different channel delays may be equal to the shift values in the used PN code. So a condition should be made on the shift values of the PN code:

(44) $$\begin{array}{c} m_{n}T_{c}\neq \tau_{l}\;\forall \;\text{values of}\;m_{n},\;{\;\tau }_{l}\in \left[0\;T_{m}\right], \end{array}$$

where *m*_*n*_ is an integer number, which represents the number of chips by which the PN code is shifted to form the *n*th code sequence in the used set of *N* spreading codes. *T*_*m*_ is the multipath delay spread and it represents the maximum delay of the longest path through which the signal propagates. One of the solutions of the inequality in (44) is

(45) $$\begin{array}{c} \max\left(m_{n}T_{c}\right)<\min\left(\tau_{l}\right). \end{array}$$

**Figure 5:**
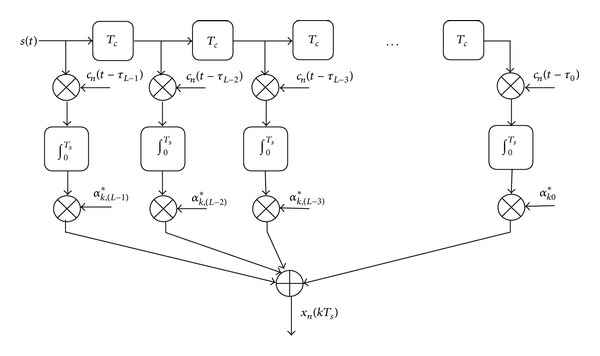
The *n*th *L*-fingers RAKE filter.

The proposed demodulator for the code-time diversity system with multipath fading channel is more complex than the demodulator shown in Figure 3 for flat fading channel. The demodulator consists of three parts too, but the first part of this demodulator is a bank of *NL*-fingers RAKE filters instead of *N* correlators. Each *L*-fingers RAKE filter correlates the received signal with *L* PN sequences. These sequences are generated from one PN code from the used set of *N* spreading PN codes according to the set of channel delays *τ*_*l*_.  Figure 5 shows the structure of the *L* figure RAKE filter that correlates the received data with the *n*th PN code in the spreading codes set.

The time resolution between the uncorrelated paths in the used RAKE receiver is *T*_*c*_. In each finger of the RAKE filter, the delayed received signal is correlated with the *n*th PN code that is shifted with an integer number of chips equal to the path delay of that finger. Conventional MRC is used to combine the output of the *L*-fingers to form the random variable *x*_*n*_(*kT*_*s*_). The output of the *l*th correlator in the *n*th RAKE filter is

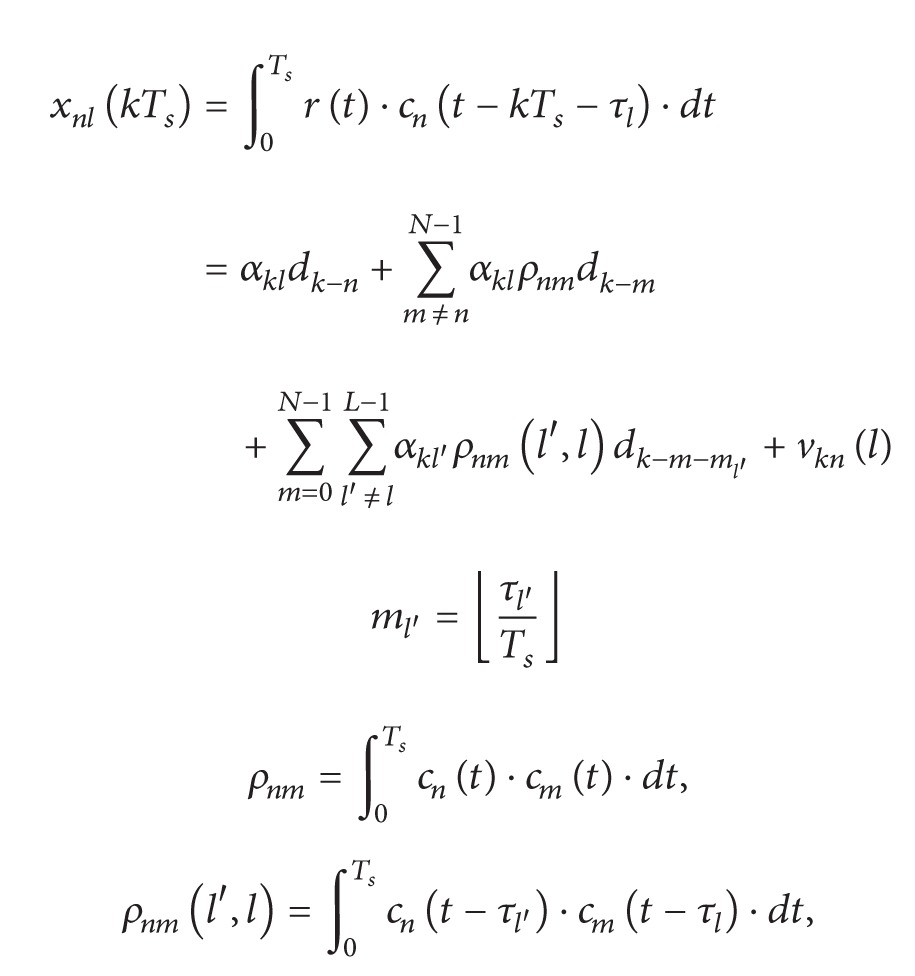
(46)

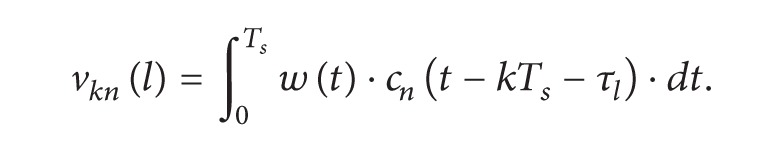
(47)
The first term in (46) is the desired signal. The second term is the ISI signal that comes from the (*N* − 1) symbols transmitted through the same symbol period. This ISI signal is due to the correlation between the used spreading codes. The third term is another ISI signals that come from the other (*L* − 1) fading paths. The last term is the noise random variable. Using (46), the output of the *n*th RAKE filter is

(48) xn(kTs)=∑l=0L−1|αkl|2dk−n+∑l=0L−1∑m≠nN−1|αkl|2ρnmdk−m +∑l=0L−1 ∑m=0N−1 ∑l′≠lL−1βkl′ρnm(l′,l)dk−m−ml′+vknβll′=αkl′·αkl∗.

**Figure 6:**
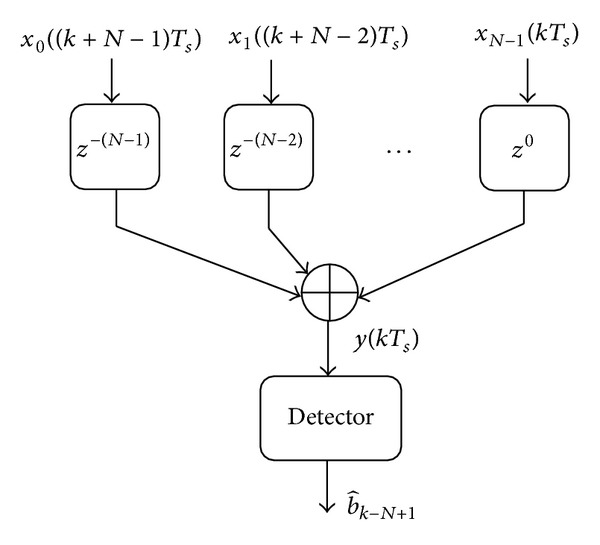
The delayed symbols combiner of the outputs of RAKE filters.

The last term *v*_*kn*_ is a Gaussian random variable with zero mean and ∑_*l*=0_^*L*−1^|*α*_*kl*_|^2^ *σ*_*w*_^2^ variance. The second part of the proposed demodulator is the delayed symbols combiner. The DSC delays the output random variable from each RAKE filter according to the index of the PN sequence used in this RAKE filter. The output of the RAKE filter with the code sequence *c*_*n*_(*t*) is delayed ((*N* − 1) − *n*) symbol periods.  Figure 6 presents the structure of DSC.

The delayed signals are finally added to form a single input to the detector. The output of the DSC in the *z*-domain can be represented by

(49) Y(z)=X0(z)z−(N−1)+X1(z)z−(N−1)+1 +X2(z)z−(N−1)+2+⋯+XN−1(z).

The output of the DSC combiner at the *k*th symbol period is represented by

(50) $$\begin{array}{c} y\left(kT_{s}\right)=\overset{N-1}{\underset{n=0}{\sum }}x_{n}\left(\left(k-\left(N-1\right)+n\right)T_{s}\right)+v_{kn}^{\prime }, \end{array}$$

(51) y(kTs)=∑n=0N−1 ∑l=0L−1|αnl(kTs)|2dk−(N−1) +∑n=0N−1 ∑l=0L−1 ∑m≠nN−1|αnl(kTs)|2ρnmdk−(N−1)+n−m +∑n=0N−1 ∑l=0L−1 ∑m=0N−1 ∑l′≠lL−1βnll′(kTs)ρnm(l′,l)×dk−(N−1)+n−m−ml′+vkn′,

(52) $$\begin{array}{c} v_{kn}^{\prime }=\overset{N-1}{\underset{n=0}{\sum }}\;\overset{L-1}{\underset{l=0}{\sum }}\beta_{nl}\left(kT_{s}\right)v_{k-(N-1)+n}\left(l\right), \end{array}$$

where *v*_*in*_′ is a Gaussian random variable with zero mean and ∑_*n*=0_^*N*−1^∑_*l*=0_^*L*−1^|*α*_*nl*_(*kT*_*s*_)|^2^ *σ*_*w*_^2^ variance. As in flat fading case, the estimated data at the output of the detector is delayed (*N* − 1) symbol periods. This delay represents the time spread at which the transmitted symbol is repeated. If the correlation between the used spreading codes is zero, the combined signal in (51) will be

(53) $$\begin{array}{c} y\left(kT_{s}\right)=\overset{N-1}{\underset{n=0}{\sum }}\;\overset{L-1}{\underset{l=0}{\sum }}{{\left|\alpha_{nl}\left(kT_{s}\right)\right|}^{2}d}_{k-(N-1)}+v_{kn}^{\prime }. \end{array}$$

The last part of the demodulator is the detector. The estimated symbol at the output of the detector is the symbol with the minimum distance to the detector input as shown in (14).

The combined signals in (53) are equivalent to that of (*L* × *N*)-branch MRC receiver. Thus, the resulting diversity order of the new code-time transmit diversity scheme with *N* spreading orthogonal codes and one transmitting and receiving antenna in frequency selective channel with *L* faded paths is equal to that of the (*L* × *N*)-branch MRC receiver scheme.

The combined signals in (53) are also similar to that of space-time MIMO system with *N* antennas at the transmitter and *L* antennas at the receiver. The proposed code-time diversity system does not use additional encoders or decoders at the transmitter or the receiver such as the space-time encoder and decoders in the space-time MIMO systems. No additional RF interface circuits or antennas are used in the code-time diversity. Spreading and dispreading circuits are the only used additional hardware. Although the code-time diversity has the disadvantage of low bandwidth efficiency due to the usage of DSSS, the extended bandwidth in DSSS increases the channel capacity and the DSSS can resist the jamming and noncochannel interference signals.

The ISI signals that appear in (51) can be eliminated or neglected if the correlation between the spreading codes is zero or very small, respectively. If spreading codes with unavoidable cross-correlation are used as PN sequences, the ISI signals can be minimized by using long codes sequences or by using linear equalizers.

To determine the probability of error in the proposed code-time diversity system in frequency selective channel, the same procedure as that used in flat fading channel case is followed. The decision variable in the detector is calculated first. Then the conditional probability of error is calculated given a fixed set of channel gains. Finally the average probability of error is calculated based on the probability density function of the decision variable. Based on (53), the decision variable for the case of orthogonal spreading codes is

(54) $$\begin{array}{c} \text{DV}=\overset{N-1}{\underset{n=0}{\sum }}\overset{L-1}{\underset{l=0}{\sum }}{{\left|\alpha_{nl}\left(kT_{s}\right)\right|}^{2}E}_{s}+v_{in}^{\prime \prime }, \end{array}$$

where *v*_*in*_′′ is a Gaussian random variable with zero mean and ∑_*n*=0_^*N*−1^∑_*l*=0_^*L*−1^|*α*_*nl*_(*kT*_*s*_)|^2^ *E*_*s*_ *σ*_*w*_^2^ variance and |*α*_*nl*_(*kT*_*s*_)|^2^ is a chi-square random variable with two degrees of freedom. The instantaneous SNR is

(55) $$\begin{array}{c} \text{SNR}=\frac{{E\left[\text{DV}\right]}^{2}}{2\;\text{var}\left[\text{DV}\right]}=\frac{E_{s}\sum_{n=0}^{N-1}\sum_{l=0}^{L-1}{\left|\alpha_{nl}\left(kT_{s}\right)\right|}^{2}}{2\sigma_{w}^{2}}. \end{array}$$

SNR in (55) is a chi-square with 2*N* × *L* degrees of freedom. The pdf of the SNR random variable is represented in (21) where *NL* replaces *N*. The average probability of error of code-time diversity system in *L*-paths Raleigh fading channel is

(56) P−QAM=  4(1−1M)(1(N−1)!SNR−NL) ×(2(M−1)3)NLΓ(NL+(1/2))π(2NL) ×  2F1(NL,2NL+12;N+1;−2(M−1)3SNR−).

In nonorthogonal spreading code case, the detector decision variable is

(57) DV=∑n=0N−1∑l=0L−1|αnl(kTs)|2Es +∑n=0N−1∑l=0L−1∑m≠nN−1|αnl(kTs)|2ρnmdk−(N−1)+n−m·dk−(N−1)∗ +∑n=0N−1∑l=0L−1∑m=0N−1∑l′≠lL−1βnll′(kTs)ρnm(l′,l)dk−(N−1)+n−m−ml′·dk−(N−1)∗+vin′′.

Following the same procedure as Rayleigh flat fading case, the instantaneous SINR is

(58) SINR(kTs)=Es(∑n=0N−1∑l=0L−1|αnl(kTs)|2)2(N−1)ρ2Es(∑n=0N−1∑l=0L−1|αnl(kTs)|4+∑n=0N−1∑l=0L−1∑l′≠lL−1|βnll′(kTs)|2)+2σw2∑n=0N−1∑l=0L−1|αnl(kTs)|2.

Numerical calculations of the pdf of the SINR random variable in (58) show that the SINR random variable can also be approximated to a Nakagami random variable as represented in Section 4.2. Following the same procedure, the average probability of symbol error of code-time diversity system in *L*-paths Rayleigh fading channel with nonorthogonal spreading codes is

(59) P−QAM=(1−1M)21−mLΓ(2mL)Γ(mL) ·(13·e((9/(32(M−1)2))·(LΩ/m))·D−2m(98(M−1)2·LΩm)+e((1/(2(M−1)2)·(LΩ/m))·D−2m(2(M−1)2·LΩm)).

If Gaussian pdf is used to approximate the pdf of SINR, the average probability of symbol error will be

(60) P−QAM=2(1−1M) ·(16e(3L/2)((3σ2)/(4(M−1)2)−(μ/(M−1)))+12e2L((σ2/(M−1)2  )−(μ/(M−1)))).

## 5. Simulations

The proposed code-time diversity system is simulated using a DSSS system. Two different spreading codes are used. Walsh codes simulate the case of orthogonal codes' set; however, PN codes simulate the case of nonorthogonal codes' set. Different number of spreading codes *N* is used to achieve transmitter diversity. The used modulation scheme is 16-QAM. The transmitted symbols rate is 5 M symbol/s. The transmitted signal carrier frequency is 10 GHz. The used process gains are 11.78 dB and 15 dB. The transmitted signal bandwidths are 150 MHz and 310 MHz according to the used spreading code and its process gain.

**Figure 7:**
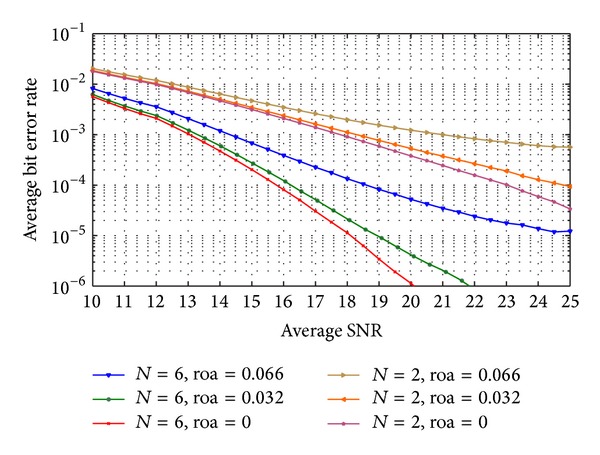
The average probability of bit error of code-time diversity system in Rayleigh flat fading channel with *N*  = 2, 6 using orthogonal and nonorthogonal spreading codes.

**Figure 8:**
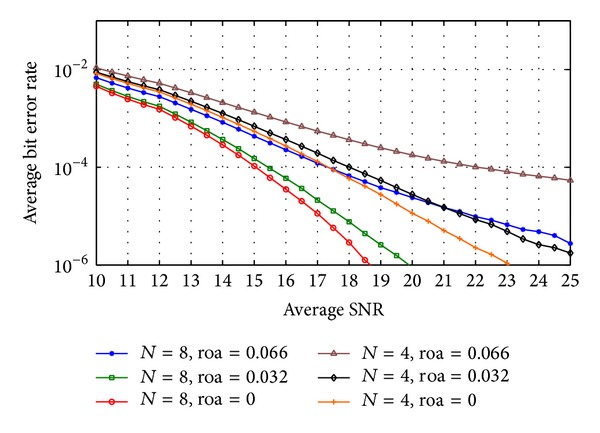
The average probability of bit error of code-time diversity system in Rayleigh flat fading channel with *N*  = 4, 8 using orthogonal and nonorthogonal spreading codes.

Figures 7 and 8 show the average probability of bit error in the received data when code-time diversity is used in Rayleigh flat fading channel. The simulated system used *N*  = 2, 4, 6, and 8 code sequences. For nonorthogonal code set, PN sequences with 31 chips period and 15 chips period are used.  Figure 7 contains the probability of error curves for *N*  = 2, 6 and Figure 8 contains the curves of *N*  = 4, 8. In orthogonal codes case, the proposed system achieved diversity gain proportional to the number of the used codes *N*. Increasing the code diversity by increasing *N* will increase the diversity order and enhance the system performance. The orthogonality between the used codes prevents the ISI from appearing. The probability of error curves of the orthogonal codes case in these figures is the same as the probability of error curves of the diversity systems in [24] using the same diversity order. The curves also realize (24) for Rayleigh flat fading channel. The figures likewise show the case of nonorthogonal codes where the ISI appeared. The ISI increases the average probability of error as shown in (39) and (40). If the code period of the used code increases, the cross-correlation between the codes pairs decreased and the average probability of error is improved.

**Figure 9:**
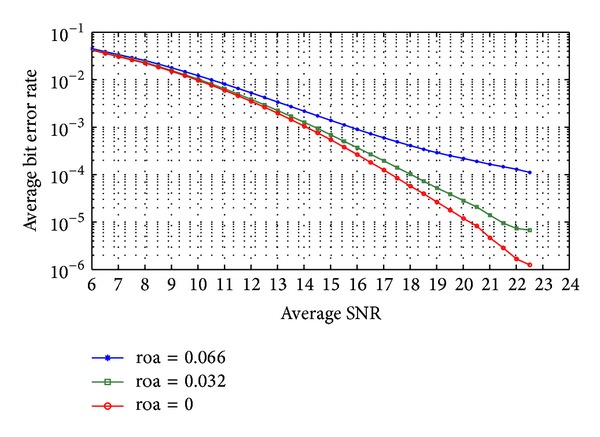
The average probability of bit error of code-time diversity system in Rayleigh frequency selective fading channel with *N* = 2 and *L* = 2, using orthogonal and nonorthogonal spreading codes.

**Figure 10:**
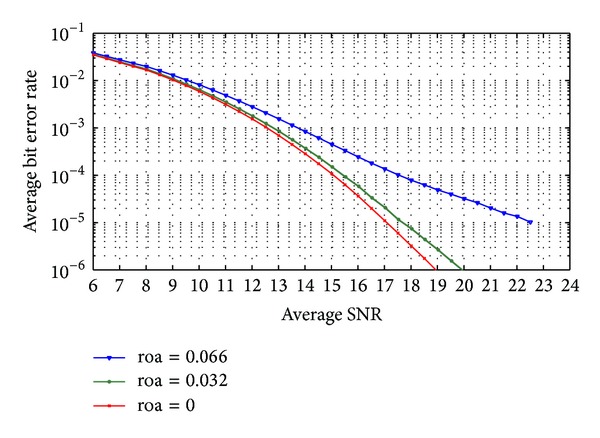
The average probability of bit error of code-time diversity system in Rayleigh frequency selective fading channel with *N* = 4 and *L* = 2, using orthogonal and nonorthogonal spreading codes.

**Figure 11:**
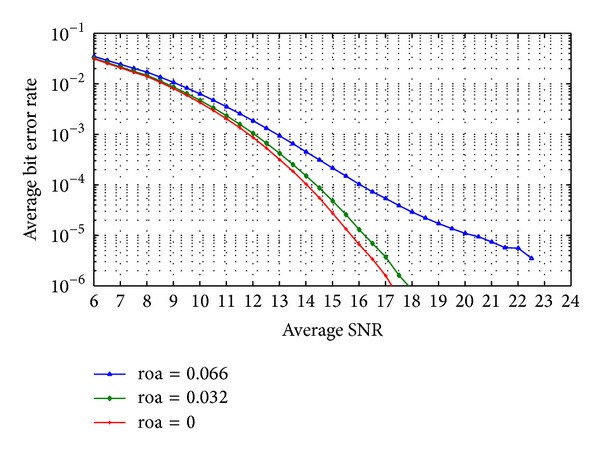
The average probability of bit error of code-time diversity system in Rayleigh frequency selective fading channel with *N* = 4 and *L* = 4, using orthogonal and nonorthogonal spreading codes.

The code-time diversity system is simulated in Rayleigh frequency selective channel with *L* = 2 and 4. As shown before, the performance of the proposed code-time diversity system in frequency selective channel is similar to the performance of the MIMO diversity system. The diversity order in the proposed system will equal the multiplication of the number of used codes (*N*) in the transmitter by the number of the signal paths (*L*) of the channel.  Figure 9 shows the average bit error rate in the received data for *N* = 2 and *L* = 2; that is, diversity order is 4. The proposed code-time diversity system is simulated using orthogonal and nonorthogonal codes. In orthogonal codes case, the average probability of error matches the values of (56) and the average probability of error in 2 × 2 MIMO system in [24]. On the other hand, the nonorthogonal codes case gives rise to ISI and the average probability of error will increase. As the code period increases, the correlation between the different codes pairs decreases and so the ISI between the successive symbols. The same results are achieved in Figures 10 and 11 for *N* = 4, *L* = 2 and *N* = 4, *L* = 4 cases, respectively. Furthermore, the performance of the simulated systems for orthogonal code case in Figures 10 and 11 is the same as the performance of 4 × 2 and 4 × 4 MIMO systems in [24], respectively.

## 6. Conclusions

The proposed code-time diversity is a diversity system suitable for direct sequence spread spectrum. The proposed diversity scheme uses single RF interface unit and single antenna at the transmitter and receiver. The proposed system achieves the benefits of diversity systems as well as the benefits of spread spectrum systems. If orthogonal spreading codes are used, the performance of the code-time diversity system is similar to the performance of the MIMO system with the same diversity order. The code-time diversity can achieve a higher diversity order than the MIMO system, which is limited with the number of the used antennas and the RF interface units. The proposed system is suitable for working in flat and frequency selective channels. The proposed system also gives a good performance if nonorthogonal codes are used as long as the cross-correlation between the used codes pairs is small enough. The paper represents mathematical derivations of the probability of error of the proposed system in nonfaded and Rayleigh faded channels for orthogonal and nonorthogonal spreading codes. The disadvantage of the proposed system is the bandwidth efficiency. This disadvantage can be enhanced if multiusers are allowed to share the same channel bandwidth with different spreading codes set.

## Appendices

### A. Definition of the Generalized Hypergeometric Function

The generalized hypergeometric function _*p*_ *F*_*q*_ has a series expansion as shown in the following equation:

(A.1)  pFq({a1,a2,…,ap  };{b1,b2,…,bq};z)  =∑k=0∞(a1)k⋯(ap)k(b1)k⋯(bq)k·zkk!.

This mathematical function is suitable for both symbolic and numerical manipulation. (*a*)_*k*_ is the Pochhammer symbol defined as

(A.2) $$\begin{array}{c} {\left(a\right)}_{k}=\frac{\Gamma \left(a+k\right)}{\Gamma \left(a\right)}. \end{array}$$

### B. The Evaluation of the Integration in (38)

Starting from (38),

(B.1) P−QAM=∫0∞PQAM·p(SINR(kTs))·dSINR=2(1−1M)2Γ(m)·(mΩ)m ×∫0∞erfc⁡(32(M−1)SINR)  ×SINR2m−1e−(m/Ω)SINR2·dSINR.

From [25], the *erfc*⁡() can be approximated to

(B.2) $$\begin{array}{c} \mathrm{erfc}\left(x\right)\approx \frac{1}{6}e^{-x^{2}}+\frac{1}{2}e^{(-4/3)x^{2}}\;\;\;\;\;\;\;\;\;\;\;\; \end{array}$$

(B.3) P−QAM =2(1−1M)2Γ(m)·(mΩ)m  ×[∫0∞16SINR2m−1e−(m/Ω)SINR2e−(a/2)SINR·dSINR+∫0∞12SINR2m−1e−(m/Ω)SINR2e−(2a/3)SINR·dSINR],

where *a* = 3/(*M* − 1).

From  3.462 in [23],

(B.4) $$\begin{array}{c} \overset{\infty }{\underset{0}{\int }}x^{v-1}\cdot e^{-Bx^{2}}\cdot e^{-\gamma x}\cdot dx={\left(2B\right)}^{-v/2}\Gamma \left(v\right)e^{\gamma^{2}/8B}D_{-v}\left(\frac{\gamma }{\sqrt{2B}}\right). \end{array}$$

Referring to (B.3), *v* = 2*m*, *B* = 2/*Ω*, and *γ* = *a*/2 for the first integral and  *γ* = 2*a*/3  for the second one.

By substituting (B.4) into (B.3), the integration can be solved and the final value of the average probability of error can be represented as

(B.5) P−QAM=(1−1M)21−mΓ(2m)Γ(m) ·(13·e((9/(32(M−1)2).(Ω/m))·D−2m×(98(M−1)2·Ωm)+e((1/(2(M−1)2)·(Ω/m))·D−2m(2(M−1)2·Ωm)).
